# Three years of insecticide resistance monitoring in *Anopheles gambiae* in Burkina Faso: resistance on the rise?

**DOI:** 10.1186/1475-2875-11-232

**Published:** 2012-07-16

**Authors:** Athanase Badolo, Alphonse Traore, Christopher M Jones, Antoine Sanou, Lori Flood, Wamdaogo M Guelbeogo, Hilary Ranson, N’Fale Sagnon

**Affiliations:** 1 Centre National de Recherche et de Formation sur le Paludisme, BP 2208, Ouagadougou 01, Burkina Faso; 2 Vector Group, Liverpool School of Tropical Medicine, Pembroke Place, Liverpool, L3 5QA, UK; 3 Université de Ouagadougou, BP 7021, Ouagadougou 03, Burkina Faso

**Keywords:** Malaria, *Anopheles gambiae*, *kdr*, Insecticide, Resistance, *1014F*, *1014S*, *ace-1*^*R*^

## Abstract

**Background and methods:**

A longitudinal *Anopheles gambiae s.l.* insecticide-resistance monitoring programme was established in four sentinel sites in Burkina Faso. For three years, between 2008 and 2010, WHO diagnostic dose assays were used to measure the prevalence of resistance to all the major classes of insecticides at the beginning and end of the malaria transmission season. Species identification and genotyping for target site mutations was also performed and the sporozoite rate in adults determined.

**Results:**

At the onset of the study, resistance to DDT and pyrethroids was already prevalent in *An. gambiae s.l.* from the south-west of the country but mosquitoes from the two sites in central Burkina Faso were largely susceptible. Within three years, DDT and permethrin resistance was established in all four sites. Carbamate and organophosphate resistance remains relatively rare and largely confined to the south-western areas although a small number of bendiocarb survivors were found in all sites by the final round of monitoring. The *ace-1*^*R*^ target site resistance allele was present in all localities and its frequency exceeded 20% in 2010 in two of the sites. The frequency of the *1014F kdr* mutation increased throughout the three years and by 2010, the frequency of *1014F* in all sites combined was 0.02 in *Anopheles arabiensis*, 0.56 in *An. gambiae* M form and 0.96 in *An. gambiae* S form. This frequency did not differ significantly between the sites. The *1014S kdr* allele was only found in *An. arabiensis* but its frequency increased significantly throughout the study (P = 0.0003) and in 2010 the *1014S* allele frequency was 0.08 in *An. arabiensis.* Maximum sporozoite rates (12%) were observed in Soumousso in 2009 and the difference between sites is significant for each year.

**Conclusion:**

Pyrethroid and DDT resistance is now established in *An. gambiae s.l.* throughout Burkina Faso. Results from diagnostic dose assays are highly variable within and between rounds of testing, and hence it is important that resistance monitoring is carried out on more than one occasion before decisions on insecticide procurement for vector control are made. The presence of *1014S* in *An. gambiae s.l.*, in addition to *1014F*, is not unexpected given the recent report of *1014S* in Benin but highlights the importance of monitoring for both mutations throughout the continent. Future research must now focus on the impact that this resistance is having on malaria control in Burkina Faso.

## Background

Insecticide resistance monitoring should be an essential component of any malaria vector control programme. Vector control activities have been dramatically scaled up in recent years across much of sub-Saharan Africa with a concomitant reduction in malaria cases [1]. In order to maintain this success, insecticide resistance management plans must be incorporated into National Malaria Control Programmes (NMCP). Burkina Faso distributed approximately eight million long-lasting insecticide-treated nets (LLINs) in the past 16 months and indoor residual spraying (IRS) with bendiocarb has been ongoing since 2009 in the south-western part of the country. Although several published papers have indicated the presence of resistance, or suspected resistance, to all the major classes of insecticides used in malaria control in some sites in Burkina Faso [2-6], routine insecticide resistance monitoring is not yet integrated in the NMCP.

The pattern of emergence of insecticide resistance in Burkina Faso mirrors that in many other countries, with small foci of resistance expanding rapidly in the past decade. Resistance to pyrethroids was detected in the south-western region of Burkina Faso as early as 1999 [7,8] and remained restricted to this region, which is the site of the majority of the cotton growing and other commercial agriculture, when next surveyed in 2006 [9]. The frequency and distribution of the target site mutation, *1014F*, conferring resistance to pyrethroids and DDT, increased during this time period. At the beginning of the century, *1014F* was found only in the south-western region and largely restricted to the *Anopheles gambiae* S form [10] but by 2006, *1014F* was found in *An. gambiae* M and S form and *Anopheles arabiensis* and had spread to the central region of the country.

The *ace-1*^*R*^ mutation, associated with organophosphate and carbamate resistance, has also been detected in *An. gambiae s.l.* in the south-western region of Burkina Faso [3,5].

The WHO/TDR network on insecticide resistance in African malaria vectors was established in 2008 and ran for three years in four countries: Benin, Burkina Faso, Chad and Sudan [6]. One of the major objectives was to improve the monitoring of insecticide resistance in areas where malaria control programmes are being implemented. Here, results from the three-year study in Burkina Faso are reported. They demonstrate that pyrethroid resistance is now present in all four sentinel sites and that both the *1014F* and *1014S kdr* alleles are firmly established in *An. gambiae s.l.* throughout the country.

## Methods

### Study area and insecticide bio-assays

**Figure 1:**
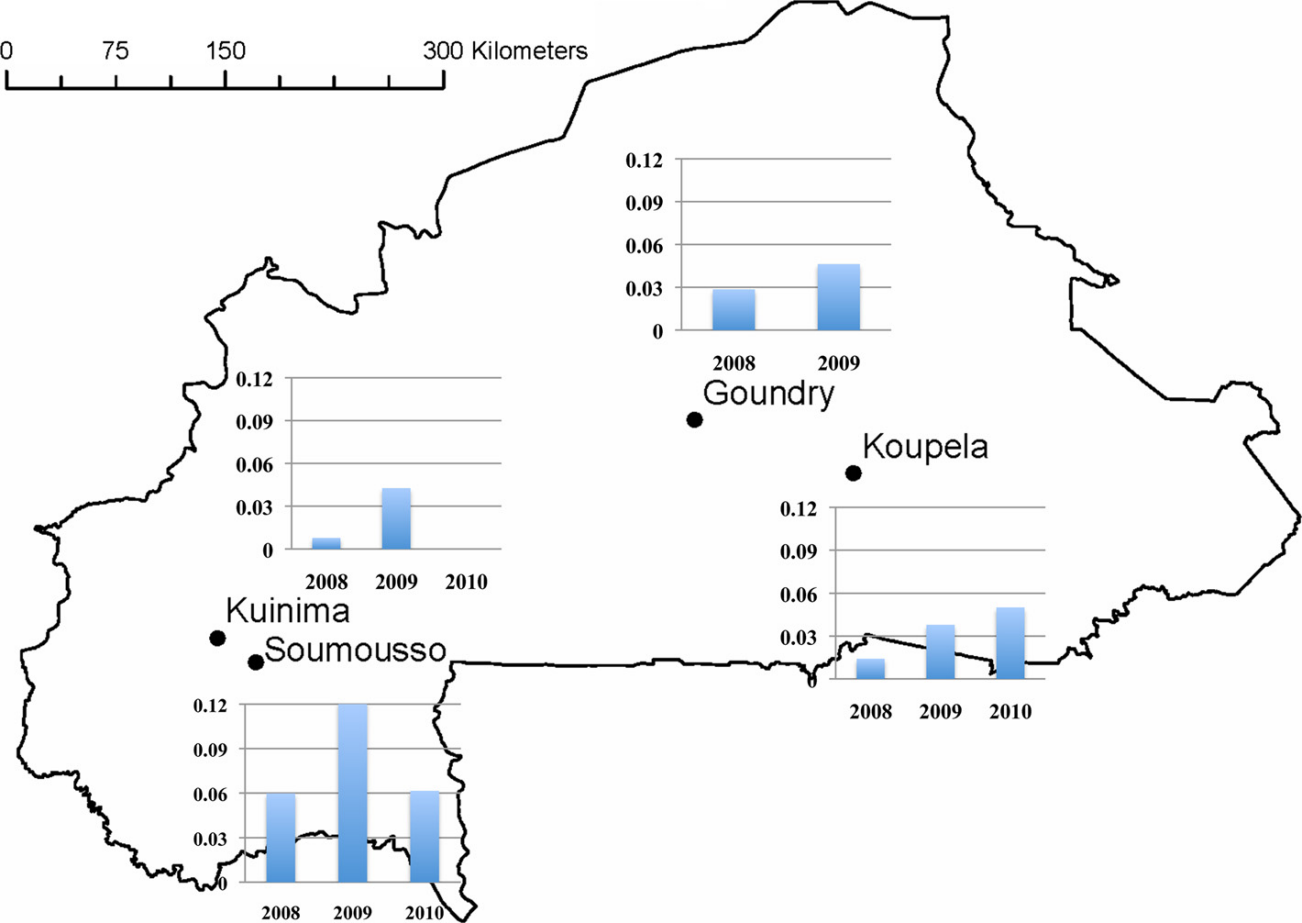
Map showing geographical locations of the study sites and the percentage of adult mosquitoes positive for sporozoites (Y axis) during years of collection (X axis) of the study (data for Goundry 2010 not available).

Mosquito larvae were collected in four localities throughout the country (Figure 1) according to the agro-ecological areas and the intensity of insecticide use in agriculture or for public health purpose.

Goundry (N 12° 30, W 01° 20) is in the Sahelian zone, in the south of Ouagadougou, 30 km far from the capital city. There is a low insecticide usage for agricultural purposes and this area was not targeted by the NMCP for LLIN distribution before 2010.

Koupela (N 12° 10, W 00° 21) is in the Sudan Sahelian zone, approximately 130 km east of the capital city, Ouagadougou. This village is characterized by gardening, rice cultivation and food crops. Extensive distribution of LLINs has been completed by the NMCP and other social partners.

Kuinima (N 11° 09, W 04° 17) is in the Sudan Savannah zone, south-west Burkina Faso, approximately 4 km from Bobo Dioulasso. This site is characterized by food crops and gardening where insecticides are used to protect vegetable crops from insect damage. No large scale distribution of LLINs was carried out pre-2010.

Soumousso is a rural village located (N 11° 00, W 04° 03) in the Sudan Savannah zone, in the south-western part of the country. It is situated 38 km from Bobo Dioulasso and is characterized mainly by rice and cotton cultivation. Pyrethroids, organophosphates and carbamates are widely used to control agricultural pests. As in Kunima, this area was not targeted for LLIN distribution before 2010.

Collections were made twice each year, at the end of July and end of October, from 2008 to 2010. Mosquitoes were collected as larvae and reared to adults for insecticide bio-assays as described in Ranson *et al.*[6].

### Species and molecular form identification

Genomic DNA was extracted from individual mosquitoes using a modification of the Livak protocol [11]. In 2008 and 2009, a random sample of between 36 and 44 mosquitoes from the bio-assay controls, plus all the survivors of insecticide and an equal number of dead specimens were identified to species and molecular forms of *An. gambiae s.s.* using the methods described by Scott *et al.*[12] and Favia *et al.*[13] respectively. In 2010, the protocol was altered so that, rather than extracting DNA from the control bio-assays, two test bio-assays from each of the permethrin, deltamethrin and DDT bio-assays were selected at random from each site in each collection round and all survivors and dead were used for DNA extraction. Thus in 2008 and 2009, the sample size for species identification was between 50 and 70 per site per collection round, whereas in 2010 this was increased to over 150. In 2010, the species identification was performed at the Liverpool School of Tropical Medicine and the SINE method was used [14].

### Detection of *kdr* alleles

Mosquitoes from the control bio-assay (or the entire subset from selected bio-assays for 2010) were used to determine the frequency of the *1014F* and *1014S* alleles in the general population. For these assays the TaqMan genotyping method was used [15]. To determine the association between *kdr* genotype and the probability of surviving the WHO bio-assay, the surviving mosquitoes from the permethrin, deltamethrin and DDT bio-assays, plus an equal number of dead mosquitoes were genotyped using the allele specific PCR method [16,17].

### *Ace-1*^*R*^ mutation detection

Control mosquitoes from 2008 (n = 107) were genotyped for the *ace-1*^*R*^ mutations using the protocol of Weill *et al.*[18]. In 2010, the mosquitoes used in fenitrothion bio-assays (all survivors and dead from a random selection of bio-assay experiments) were used for *ace-1*^*R*^ genotyping (n = 661).

### Sporozoite detection

Adult mosquitoes were collected from the four sentinel sites using a mixture of pit traps, exit traps and indoor pyrethroid spray catches. The heads and thorax of the female mosquitoes were tested by ELISA for the presence of *Plasmodium falciparum* circumsporozoite protein (CSP) [19].

### Statistical analysis

Bio-assay data were considered for each insecticide, locality, collection round and year. Mortality was calculated as the percentage of individuals that died within 24 h of exposure. Bio-assay outcomes were assessed according to WHO [20]. Those with an overall mortality > =98% were considered susceptible, those with mortality <98% but >80% were considered potentially resistant, and, those with mortality <80% were strongly suspected to be resistant. The 95% confidence limits of the mortality were calculated using Microsoft Excel and bio-assay mortalities from the first (2008) and third (2010) years were compared using Z proportion tests. For this analysis, data from both rounds of testing in each year were pooled.

Temporal and spatial variations in members of *An. gambiae* species complex were investigated for each site using Fisher Exact test.

The chances of surviving DDT or permethrin diagnostic doses for each species/molecular form were analysed using a two-tailed Fisher exact test to compare the probability of survival in alive and dead mosquitoes between pairs of species.

The change in the *1014F, 1014S* and *ace-1*^*R*^ frequencies were compared between sites and between years using Fisher exact test with significance level set at 0.05. The correlation between *1014F* genotype and survival to insecticide was determined using χ^2^ tests.

The *An. gambiae* sporozoite infection rate and 95% confidence limits were calculated in each site. The difference in the infection of mosquitoes has been compared using Z test to compare proportions between sites and between years for the same site.

## Results

### Insecticide bio-assays

**Figure 2:**
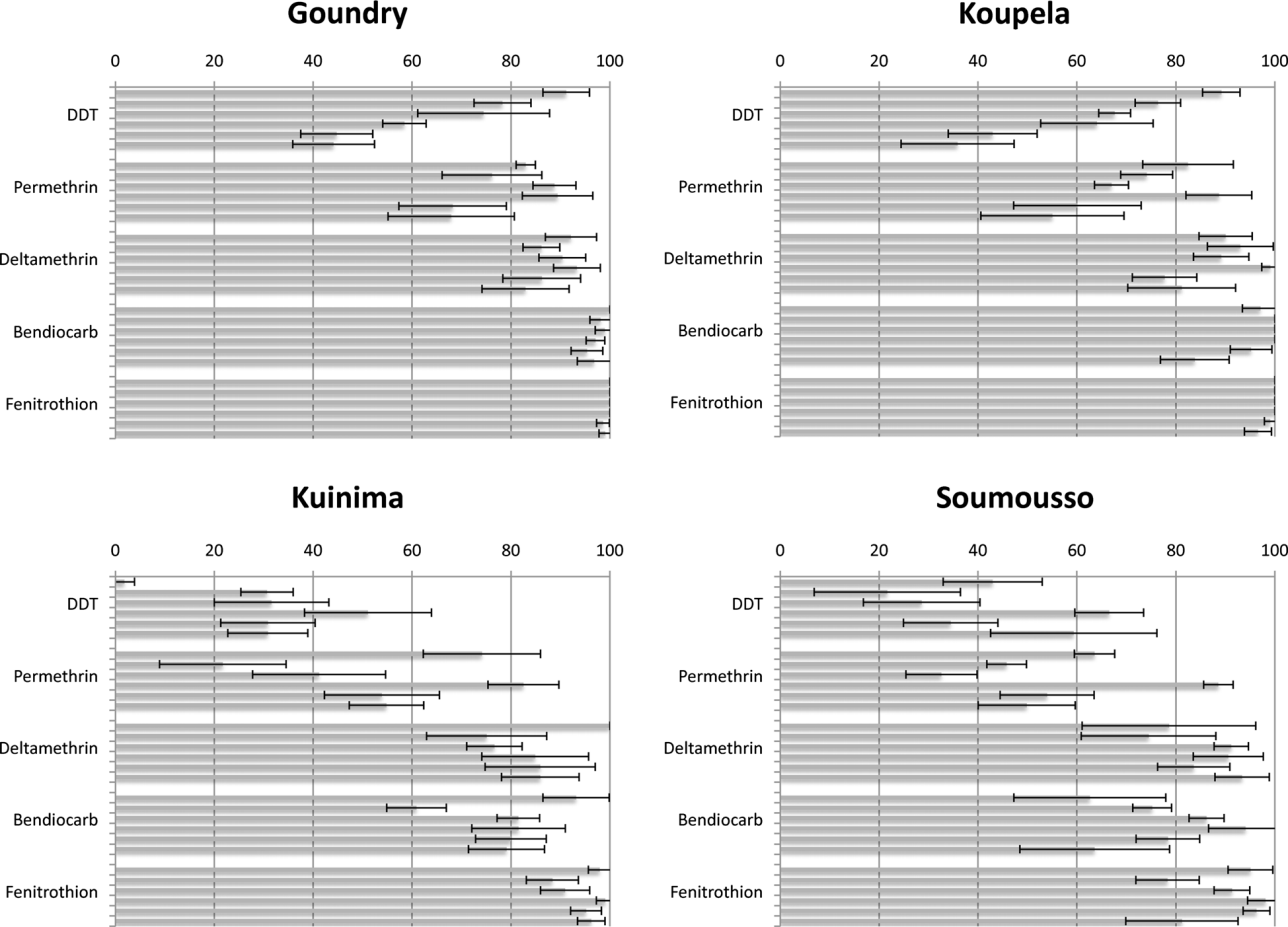
**Insecticide bio-assay results for *Anopheles gambiae s.l *from 2008–2010 in two rounds of monitoring in four sentinel sites in Burkina Faso (data from 2008 have been already published **[6]**and are included for the purpose of comparison).** The percentage mortalities 24 hours following a one-hour exposure to the WHO diagnostic dose of insecticide (with 95 % CI) are shown. The minimum sample size for each test was 100 non-blood fed female mosquitoes, three to five days old.

The results of the insecticide bio-assays are shown in Figure 2 (data from 2008 have been published previously [6], but are included here for comparison). Using the WHO definitions of resistance, the two populations from the arid savannah zone in central Burkina Faso, Goundry and Koupela, were either fully susceptible or showed a very low prevalence of resistance to all five insecticides tested at the start of the study in 2008. Three years later, resistance to DDT and permethrin was established in these populations. In the Sudan savannah region in the south-west of the country (sites Kuinima and Soumousso), low mortality with DDT and permethrin were observed throughout the study. The deltamethrin bio-assays fluctuated around the resistance cut-off of 80% throughout the three years in all four sites but with most data sets not meeting the WHO definition of resistance.

Survival after exposure to the carbamate bendiocarb also fluctuated around the 80% level in Soumousso and Kuinima. Higher mortality rates were obtained in Goundry and Koupela but some mosquitoes were surviving the diagnostic dose in these sites at the end of the study. Exposure to the organophosphate, fenitrothion resulted in near 100% mortality in the savannah zone but there was some indication of resistance developing in the south-west of the country.

DDT mortality in Goundry and Koupela significantly decreased throughout the study while it increased in Kuinima and remained unchanged in Kuinima and Soumousso (Table 1). There was no significant change in the deltamethrin mortality except for Koupela where the mortality decreased. Bendiocarb mortality decreased significantly between the beginning and end of the study for Goundry and Koupela sites, while fenitrothion mortality increased significantly in one site, Soumousso (Table 1). The difference in mortality rate between sites for each year and each insecticide is statistically significant except for deltamethrin in 2010 (Table 1).

**Table 1:** **Insecticide bio-assay results for *Anopheles gambiae s.l. *in 2008 and 2010 in four sentinel sites in Burkina Faso (data from the two rounds in each year have been pooled for this analysis)**

**Insecticide**		**2008** **% mortality (95 % CI)**	**2010 % mortality (95 % CI)**	**P value (2008*vs* 2010)**
DDT	Goundry	85 (79 – 91)	45 (37 – 52)	<0.001
	Koupela	83 (77 – 88)	43 (34 – 52)	<0.001
	Kuinima	16 (5 – 27)	31 (21 – 40)	1
	Soumousso	32 (21 – 44)	34 (25 – 44)	0.620
	P value (between sites)	<0.001	0.001	
Permethrin	Goundry	80 (74–85)	68 (57 – 79)	0.003
	Koupela	78 (72 – 84)	60 (47 – 73)	<0.001
	Kuinima	48 (27 – 69)	54 (42 – 66)	0.879
	Soumousso	55 (48 – 62)	54 (44 – 63)	0.464
	P value (between sites)	<0.001	0.013	
Deltamethrin	Goundry	89 (85 – 93)	86 (78 – 94)	0.180
	Koupela	92 (87 – 96)	78 (71 – 84)	<0.001
	Kuinima	88 (77 – 98)	86 (75 – 97)	0.287
	Soumousso	77 (66 – 87)	84 (76 – 91)	0.967
	P value (between sites)	<0.001	0.056	
Bendiocarb	Goundry	99 (98 – 100)	95 (92 – 99)	0.010
	Koupela	99 (97 – 101)	95 (91 – 99)	0.027
	Kuinima	77 (64 – 90)	80 (73 – 87)	0.776
	Soumousso	69 (60 – 78)	78 (72 – 85)	0.989
	P value (between sites)	<0.001	<0.001	
Fenitrothion	Goundry	100 ( − )	99 (97 – 100)	0.248
	Koupela	100 ( − )	99 (98 – 100)	0.499
	Kuinima	93 (89 – 98)	95 (92 – 98)	0.402
	Soumousso	87 (79 – 94)	96 (94 – 99)	0.001
	P value (between sites)	<0.001	0.050	

Pre-exposure to the synergist, piperonyl butoxide largely restored susceptibility to pyrethroids when tested in 2010 (Additional file 1) with mortality rates ranging from 91.3% in Kuinima to 98.1% in Goundry. Full susceptibility to deltamethrin was restored for the two sites tested (Goundry and Koupela).

### Spatial and temporal distribution of *Anopheles gambiae* complex members

**Figure 3:**
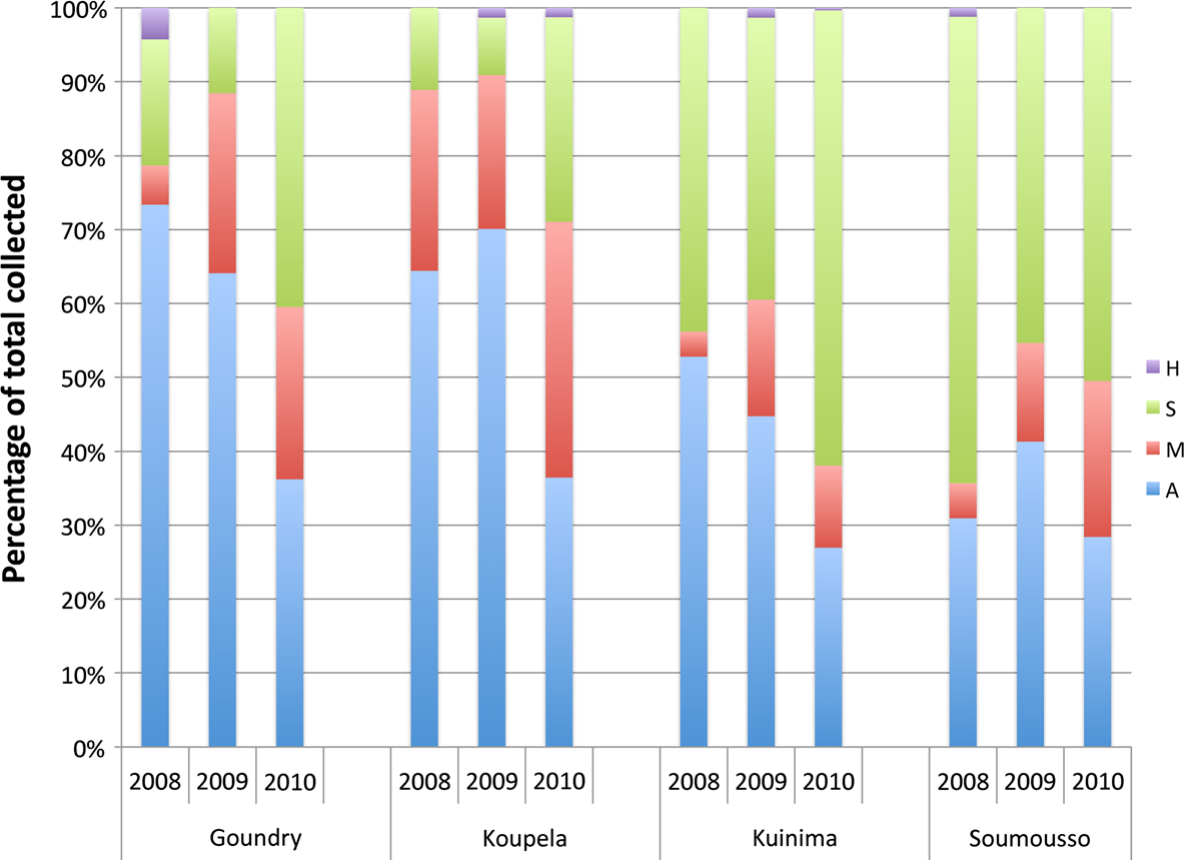
**Species identification within *Anopheles gambiae s.l. *for the four sentinel sites in 2008–2010.** Data are presented as proportions of the total for each species, *An. arabiensis* (A), *An. gambiae* M form (M), *An. gambiae* S form (S) and the hybrid of M and S forms (H), by year and by site. Sample sizes are minimum of 75 per site for 2008 and 2009 and a minimum of 309 for 2010.

A total of 5,541 *An. gambiae* complex mosquitoes from larval collection were identified to species and molecular form by PCR. Only control samples from each year (2,644 in total) were used for spatial and temporal distribution. The results are summarized in Figure 3 and Additional file 2. In 2008 and 2009, *An. arabiensis* was the most abundant species in the central sites of Goundry and Koupela and *An. gambiae s.s.* was more abundant in the south-western sites of Kuinima and Soumousso. However, by 2010, the proportion of *An. arabiensis* had fallen to less than one third in all four sites. The number of individuals with M/S hybrid patterns detected was low throughout the three years, around 1% in all the sites with largest confidence limits in Goundry (0-4%).

Within *An. gambiae* s.s., the S form dominated in Kuinima and Soumousso in all years, whereas the relative frequency of the M and S forms fluctuated more widely in Koupela and Kuinima. In 2008 and 2009 significant differences in species composition were observed between the two rounds of testing in all sites except in Goundry (2008) and Koupela and Kuinima (2009) (P < 0.05). However, when the sample size was increased in 2010 (n = 326 in 2008, 306 in 2009 and 2012 in 2010), the only significant difference (P = 0.000) was observed in Koupela, where a higher proportion on M form *An. gambiae* were present early in the transmission season than at the end of the season (Additional file 2).

**Figure 4:**
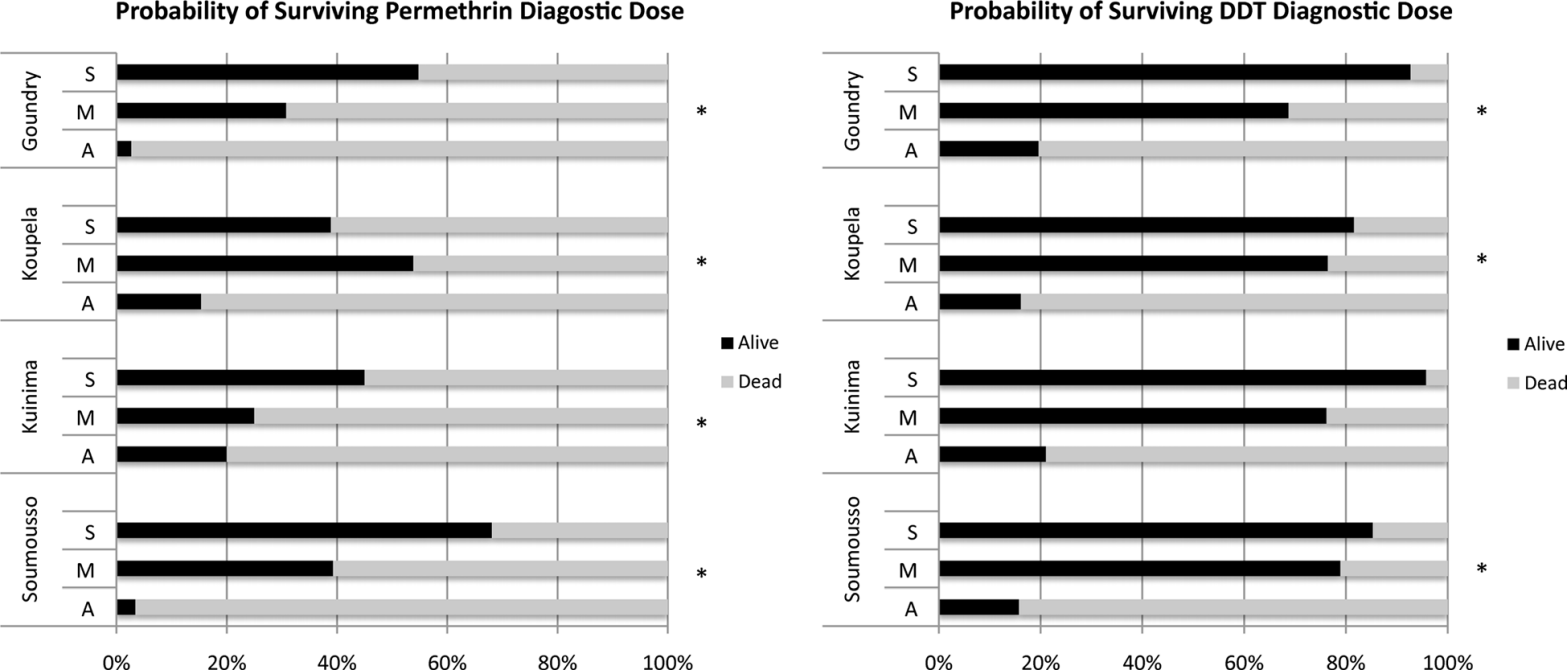
**The percentage survival of *Anopheles gambiae s.l. *species/form 24 hours following exposure to DDT or permethrin.** Data for 2010 and are stratified by species for each sentinel site. Statistical comparison between molecular forms and localities were performed using Z-test with significant differences (P < 0.05) showed in the figure (*).

The change in sampling strategy for determining the species distribution in 2010 meant it was possible to use the same sample set to determine the probability of surviving the diagnostic dose for each species in each site (Figure 4). The M and S form mosquitoes are significantly more likely to survive permethrin or DDT exposure than *An. arabiensis* (P < 0.05) except for the site of Kuinima where no significant difference has been observed between the M form and *An. arabiensis* for permethrin exposure (P =0.072 and 1 respectively for M and S compared to *An. arabiensis*). The S form exhibited higher survival rate to DDT compared to the M form in Goundry and Kuinima and only in Soumousso for permethrin.

The probability of surviving permethrin exposure for each of the three species/forms did not differ significantly between sites (S form P = 0.069, M form P = 0.337, *An. arabiensis* P = 0.053). Similarly no difference is observed between sites for probability of surviving DDT exposure (P = 0.125 for S-form, P = 0.921 for the M-form, P = 0.940).

### Frequency and distribution of *1014F* and *1014S kdr* alleles in Burkina Faso

**Figure 5:**
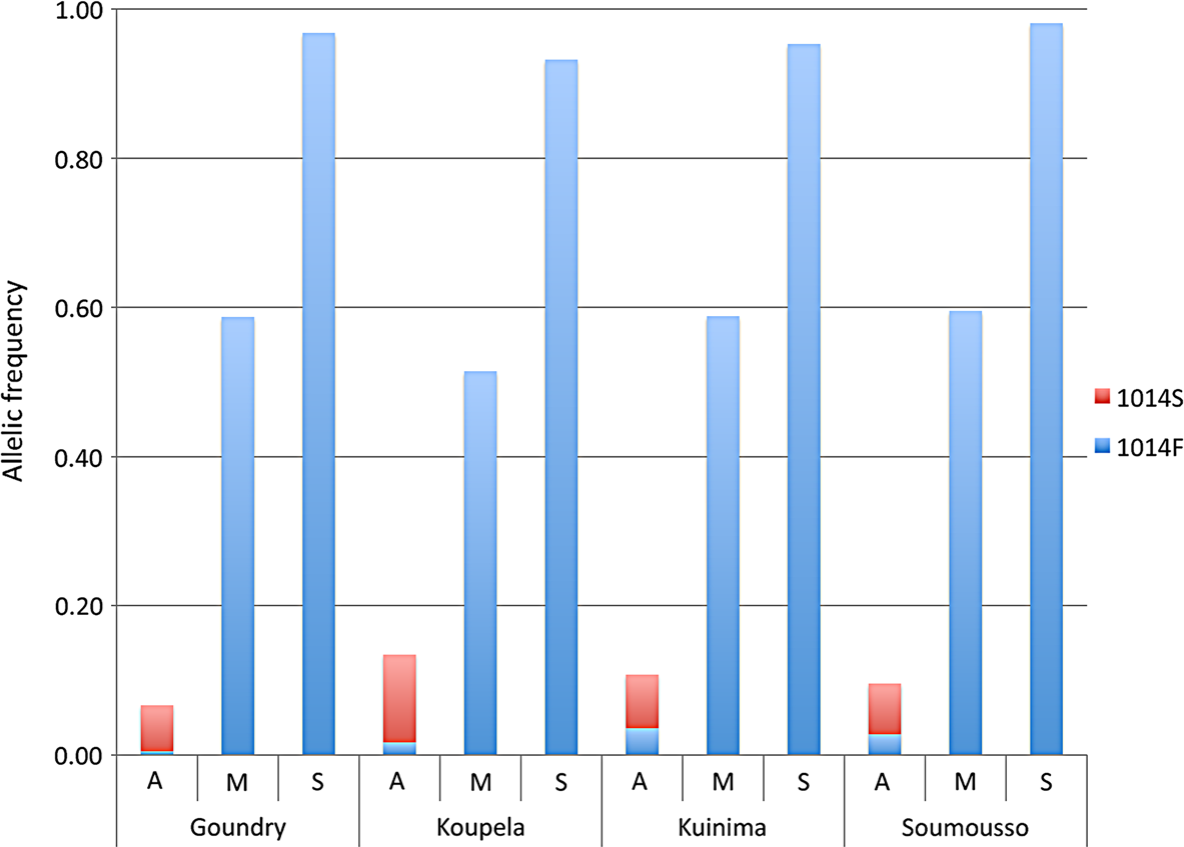
**The allele frequencies of *1014F *(blue) and *1014S *(red) by species and site.** No statistical difference is observed in the allelic frequencies between sites for each of the species/molecular form for the *1014F* mutation (P > 0.08). Differences between sites are significant when comparing for *1014S* allele in *Anopheles arabiensis.*

The frequency of the *1014F* and *1014S* alleles in the ‘control’ bio-assay populations is shown in Table 2 and allele frequencies for 2010 are shown in Figure 5.

**Table 2:** **The frequency of *1014F *and *1014S kdr *alleles in *Anopheles gambiae *M form (M), S form (S), M and S hybrids (H) and *Anopheles arabiensis *(B) in four sentinel sites in Burkina Faso, from 2008–2010**

		**2008**			**2009**			**2010**				
**Locality**	**Species**	**N**	**Freq*1014F***	**Freq*1014S***	**N**	**Freq*1014F***	**Freq*1014S***	**N**	**Freq*1014F***	**Freq*1014S***	**p value*1014F***	**p value*1014S***
Goundry	B	45	0.01	0.01	44	0.02	0.07	111	0.01	0.06	0.261	0.118
	H	3	0.50	-	0	-	-	0	-	-	-	-
	M	0	-	-	15	0.39	0.00	70	0.58	0.00	0.220	-
	S	12	0.58	0.00	5	1.00	0.00	125	0.97	0.00	0.001	-
Koupela	B	30	0.00	0.00	42	0.05	0.02	115	0.02	0.12	0.124	0.000
	H	0	-	-	0	-	-	4	0.88	-	1	
	M	14	0.36	0.00	15	0.23	0.00	107	0.51	0.00	0.056	
	S	6	0.92	0.00	3	0.83	-	89	0.92	0.00	0.348	
Kuinima	B	37	0.04	0.05	28	0.02	0.04	84	0.04	0.07	1	0.879
	H	0	-	-	1	1.00	0.00	1	1.00	-	1.00	-
	M	3	0.33	0.00	8	0.69	0.00	35	0.59	0.00	0.6845	-
	S	36	0.88	0.00	24	0.96	0.00	192	0.95	0.00	0.2437	-
Soumousso	B	16	0.00	0.00	24	0.50	0.02	89	0.03	0.07	0.6672	0.217
	H	1	1.00	0.00	0	-	-	0	-	-		-
	M	4	0.50	0.00	9	0.33	0.00	63	0.60	0.00	0.05451	-
	S	51	0.86	0.00	24	1.00	0.00	158	0.98	-	0.000	-

Frequencies of *1014F* and *1014S* for the three years are compared for each molecular form in each site using Fisher exact test. (−) represents value of the data which cannot be calculated.

In 2010, the *1014F* allelic frequency for the four sites ranged from 0.92-0.98 in the S form, 0.51-0.6 in the M form and 0.01-0.04 in *An. arabiensis*. There was no significant difference in *1014 F* frequency between the four sites (P > 0.08) (Figure 5).

The *1014S* form was detected in *An. arabiensis* only. It was found at a low frequency (Table 2), (maximum allele frequency = 0.12 in 2010). The frequency increased in all sites between 2008 and 2010 although this change was only significant in Koupela.

### Relationship between *kdr* genotype and bio-assay data

The presence of the *1014F* allele is clearly linked to DDT and pyrethroid (permethrin and deltamethrin) resistance in *An. gambiae s.l.* (P < 0.001) (Table 3). The samples size for the *1014S* mutation was too low for statistical analysis. However, of the 60 *L1014S* heterozygotes, only 11 survived exposure to either pyrethroid or DDT, suggesting that a single copy of this mutation confers little protection against exposure to the WHO diagnostic doses.

**Table 3:** **Correlation between *1014F *and *1014L *genotypes and survival to insecticides**

	**LL**	**LF**	**FF**	**χ2**	**P value**
**DDT**					
Alive	24	36	201	184.1	0.000
Dead	106	14	23		
**Deltamethrin**					
Alive	6	11	45	20.6	0.000
Dead	131	40	160		
**Permethrin**					
Alive	16	14	117	75.5	0.000
Dead	115	41	84		

LL, FF represents respectively the homozygotes for 1014L and 1014F and LF represent the heterozygotes. Data from all sites and all species tested in 2010 were pooled for this analysis.

### Frequency and distribution of *ace-1*^*R*^ allele

The *ace-1*^*R*^ allele was only detected in S form *An. gambiae* from Kuinima and Soumousso in 2008 at an allele frequency of 0.2 in both sites. By 2010, this allele was found in all sites and a single M form individual with the *ace-1*^*R*^ allele was also detected in Goundry (Table 4). No *ace-1*^*R*^ alleles were detected in *An. arabiensis.* The proportion of mosquitoes containing the *ace-1*^*R*^ allele was higher in the bendiocarb or fenitrothion survivors than the dead progeny (0.03 *vs* 0.41, P = 2e −16).

**Table 4:** **Frequency (Freq) of *ace-1***^***R ***^**alleles in *Anopheles gambiae *M form (M), S form (S), M and S hybrid (H) and *Anopheles arabiensis *(B) in four sentinel sites in Burkina Faso from 2008–2010**

		**2008**		**2010**		
**Locality**	**Species**	**N**	**Freq*ace-1***^***R***^	**N**	**Freq*ace-1***^***R***^	**P value**
Goundry	B	20	0	53	0	-
	H	1	0	4	0	-
	M	5	0	17	0.03	1.000
	S	9	0	67	0.08	0.341
koupela	B	21	0	68	0	-
	H	0	-	4	0	-
	M	7	0	42	0	-
	S	4	0	42	0.12	0.562
Kuinima	B	34	0.01	64	0	0.347
	H	0	-	5	0.10	1.000
	M	6	0	8	0	1.000
	S	97	0.20	121	0.22	0.058
Soumousso	B	30	0.02	42	0	0.417
	H	1	0	2	0	-
	M	11	0.05	17	0	0.500
	S	170	0.18	120	0.21	0.222

Allelic frequencies (N) are compared using a Fisher exact test. (−) represents values the data which cannot be calculated.

### Sporozoite rates

The presence of sporozoites in the head or thorax of adult mosquitoes was determined as part of a wider study to explore the association between insecticide resistance and transmission. Analysis is ongoing but the crude sporozoite rates are presented here to indicate the relative levels of malaria transmission in each site (Figure 1 and Table 5). The highest frequency of sporozoite positive *An. gambiae s.l.* was found in Soumousso in each year with 46 of 378 (12% CI [5.9%–15.3%]) mosquitoes positive in 2009. The sporozoite infection rate differed significantly between sites and a significant difference between years was observed in the south-western sites (Kuinima and Soumousso) (Table 5).

**Table 5:** Sporozoite rates in adult collections from study sites

**Locality**	**Year**						
	**2008**		**2009**		**2010**		**P value**
	**Sporozoite rate**	**N**	**Sporozoite rate**	**N**	**Sporozoite rate**	**N**	
Goundry	0.029 [0 – 0.045]	175	0.046 [0.008 – 0,011]	151	-	-	0.395
Koupela	0.014 [0 – 0.035]	213	0.038 [0.012 – 0.051]	397	0.05 [0.041 – 0.084]	220	0.115
Kuinima	0.008 [0 – 0.008]	130	0.043 [0 – 0.067]	47	0 [0 – 0]	129	0.038
Soumousso	0.060 [0 – 0.067]	151	0.122 [0.059 – 0.153]	378	0.061 [0.062 – 0.086]	212	0.015
P value	0.025		< 0,0001		0.020		

Proportion of Circumsporozoite Protein (*CSP)-positive Anopheles gambiae s.l.,* total numbers of tested mosquitoes and P value of proportions compared between years for each study site and P values for comparison of infected mosquitoes proportions between locality for the same year. (−) represents value of the data which cannot be calculated

## Discussion

This data set provides a comprehensive picture of the changing pattern of insecticide resistance in Burkina Faso over a three-year period. The information is of clear operational importance for malaria control in Burkina Faso but, in addition, it is hoped that several of the conclusions drawn from this data set will be of value for other countries and perhaps stimulate further debate about how resistance is measured in the field.

Focusing firstly on the pattern emerging from the different sentinel sites it is clear that resistance to DDT and permethrin is now firmly established in all four sites. At the beginning of the study, high survival rates were obtained in the south-western savannah zone sites whereas no resistance was detected in the central Sahelian sites. However, at the end of three years, resistance to these insecticides had clearly been established in *An. gambiae s.l.* in all four sites and hence, the distribution of resistance has been extended. It is important to note that nothing can be concluded about the level or impact of resistance from this data set. All that can be concluded is that a greater proportion of *An. gambiae s.l.* are surviving the diagnostic dose but, as will be discussed later, this can be partially attributed to a change in the composition of the species complex in some of the sites. To address the question of whether resistance is on the rise, data on whether the mosquitoes are becoming *more* resistant, ie, able to withstand higher concentrations of insecticide or longer exposure times is needed. This information is not obtainable from diagnostic dose assays and dose or time response assays need to be performed. At present, very little information is available about the relationship between surviving exposure to the diagnostic dose assays and the efficacy of interventions such as LLINs or IRS. Thus, while diagnostic doses are very useful for comparing between sites, as resistance becomes established in populations, more information is needed in order to understand the epidemiological impact of this resistance.

A second observation that may have resonance for other African countries is the differing response of members of the *An. gambiae* complex to pyrethroids or DDT. In all four sites the S form had the best chance of surviving DDT or permethrin exposure, followed by the M form and lastly *An. arabiensis*, which was the most susceptible. In fact, by the end of the study, the chance of surviving DDT or permethrin exposure was equal for all sites when broken down into species. Hence, understanding species dynamics is essential for predicting the spread of resistance. As an example, the increase in permethrin and DDT resistance in Goundry and Koupela during the course of the study is at least partially explained by the decreased proportion of *An. arabiensis* in these sites in 2010 (Figure 3). An important implication of this observation is that, in sites where multiple members of the *An. gambiae* complex are present, resistance must be monitored at different periods throughout the transmission season to capture this variation.

Riehle *et al.*[21] reported a very high frequency of individuals showing an M/S hybrid pattern in two of the sites in the current study (Goundry and Koupela) in 2008 and 2009, and proposed that these belonged to a new subspecies, named Goundry. Interestingly, although an unexpectedly high number of hybrids were detected in Goundry in 2008 (4.8%), no M/S hybrid patterns were observed in the 387 samples genotyped from Goundry in 2009 and 2010. The overall frequency of M/S hybrids in Koupela was approximately 1% for the three years, the reason for this discrepancy warrants further investigation.

It is unclear why the prevalence of resistance should differ so much between forms considering that all adults bio-assayed were reared from sympatric larvae and hence could be expected to be under the same selective pressure. Of course, insecticide resistance is a dynamic process and it is conceivable that within a matter of years resistance will be prevalent in all members of the species complex but the differing rates at which this is emerging in different members of the *An. gambiae* complex remains unexplained.

Equally intriguing is the failure for *1014F* to become fixed in the S form of *An. gambiae* despite fluctuating around the 0.9 allele frequency for three years. Is a fitness cost counteracting the selection pressure?

The establishment of the *1014S* allele in *An. arabiensis* in West Africa has already been noted in Benin during the TDR project [22]. The first report of this allele in the *An. gambiae* complex was in *An. gambiae* s.s. in Kenya where it was found in samples collected in 1986 [17]. This allele is now present at high frequencies in *An. gambiae* s.s from Kenya, Uganda and Burundi [23-25] and at much lower frequencies in the same species in Central Africa [26,27].

In *An. arabiensis* the frequency of both the *1014S* and *1014F* alleles is generally considerably lower than in *An. gambiae*[23,27,28]. In Burkina Faso, both *kdr* alleles were present in *An. arabiensis* although at a very low frequency (highest allele frequencies observed were 0.04 for *1014F* in 2010 in Kuinima, 0.12 for *1014S* in Koupela in the same year). Preliminary analysis of intron data (J. Pinto, unpublished data) suggest that the *1014S* allele in *An. arabiensis* in Burkina Faso has an independent origin and has not arisen by introgression from *An. gambiae*, contrary to the prediction in Kenya [23]. The source of selection pressure for *1014S* and *1014F* may differ and perhaps the former allele is more closely associated with DDT selection than pyrethroids [17]. The sample size in the current study was insufficient to establish phenotype: genotype correlations for the *1014S* allele. However, recent functional characterization of the alternative sodium channel alleles supports the importance of DDT in selecting for *1014S* by demonstrating that the *L1014S* substitution had a much greater impact on sensitivity to DDT than to pyrethroids [29].

The very high probability of surviving permethrin or DDT exposure in *An. gambiae s.s.* in Burkina Faso is at least partially explained by the high frequency of *1014F* in this species. However, undoubtedly other resistance mechanisms are contributing towards the resistance phenotype. Indeed, pre-exposure to the synergist PBO for one hour increased permethrin mortality to >90% in all sites when tested in 2010 (data not shown). This is strongly indicative of metabolic resistance mechanisms and work is ongoing to investigate this further.

The expanding geographical range and prevalence of pyrethroid resistance in Burkina Faso may impact on the efficacy of malaria control activities. There is already limited evidence that the level of resistance to pyrethroids in Soumousso is impacting on the efficacy of LLINS in use in the field [30]. In light of this threat, the NMCP in Burkina Faso has elected to use bendiocarb for the IRS campaign, which began two years ago. Unfortunately, the *ace-1*^*R*^ allele, which is associated with resistance to this carbamate insecticide, is already present in *An gambiae* s.s and appears to be spreading rapidly throughout the country. Although the bio-assay data do not indicate high frequencies of bendiocarb resistance at present, this situation should be closely monitored.

## Competing interests

The authors declare that they have no competing interests.

## Authors’ contributions

HR and NFS conceived and designed the study. AB, NFS and HR drafted the manuscript. AB and CMJ analysed the data. AB, AT, CMJ, GMW, AS, LF oversaw field collections, bio-assays and conducted laboratory work. All authors read, corrected and approved the final manuscript.

## Supplementary Material

Additional file 1 **Pyrethroid bio-assay results for *Anopheles gambiae s.l. *in four localities after exposure to PBO 4%.** An. *gambiae* mosquitoes, collected from four sentinel sites in 2010 have been exposed to PBO 4% for xxmin, prior to an exposure deltamethrin Permethrin for 1 hour. This table presents the mortality and standard error for each insecticide and locality. Click here for file

Additional file 2 **Species composition of *An gambiae s.l *between the mid and end of the malaria transmission season.** The data provided represent the statistical comparison of *An. gambiae* complex species and molecular forms composition between the mid and end of malaria transmission season during the three years of the study in the four localities. *An. gambiae* M form (M), *An. gambiae* S form (S), hybrid of S and M form, (H) and *An. arabiensis* (B) proportions are given by collection round, the mean and confidence limits (CL) of the proportion are given for each species and locality. The total number of mosquitoes PCR-tested is given for each round (R1, R2) and the P value comparing species composition between rounds is given. Click here for file

## References

[B1] World Health Organization World Malaria Report 2010 137

[B2] Dabire KR Diabate A Djogbenou L Ouari A N'Guessan R Ouedraogo JB Hougard JM Chandre F Baldet T Dynamics of multiple insecticide resistance in the malaria vector Anopheles gambiae in a rice growing area in South-Western Burkina Faso Malar J 2008 7 188 10.1186/1475-2875-7-188 18817564 PMC2564969

[B3] Dabire KR Diabate A Namontougou M Djogbenou L Kengne P Simard F Bass C Baldet T Distribution of insensitive acetylcholinesterase (ace-1R) in Anopheles gambiae s.l. populations from Burkina Faso (West Africa) Trop Med Int Health 2009 14 396 403 10.1111/j.1365-3156.2009.02243.x 19254231

[B4] Diabate A Brengues C Baldet T Dabire KR Hougard JM Akogbeto M Kengne P Simard F Guillet P Hemingway J Chandre F The spread of the Leu-Phe kdr mutation through Anopheles gambiae complex in Burkina Faso: genetic introgression and de novo phenomena Trop Med Int Health 2004 9 1267 1273 10.1111/j.1365-3156.2004.01336.x 15598258

[B5] Djogbenou L Dabire R Diabate A Kengne P Akogbeto M Hougard JM Chandre F Identification and geographic distribution of the ACE-1R mutation in the malaria vector Anopheles gambiae in south-western Burkina Faso, West Africa Am J Trop Med Hyg 2008 78 298 302 18256433

[B6] Ranson H Abdallah H Badolo A Guelbeogo WM Kerah-Hinzoumbe C Yangalbe-Kalnone E Sagnon N Simard F Coetzee M Insecticide resistance in Anopheles gambiae: data from the first year of a multi-country study highlight the extent of the problem Malar J 2009 8 299 10.1186/1475-2875-8-299 20015411 PMC2804687

[B7] Diabate A Baldet T Chandre F Akoobeto M Guiguemde TR Darriet F Brengues C Guillet P Hemingway J Small GJ Hougard JM The role of agricultural use of insecticides in resistance to pyrethroids inAnopheles gambiaes.l. in Burkina Faso Am J Trop Med Hyg 2002 67 617 622 12518852 10.4269/ajtmh.2002.67.617

[B8] Chandre F Manguin S Brengues C Dossou Yovo J Darriet F Diabate A Carnevale P Guillet P Current distribution of a pyrethroid resistance gene (kdr) inAnopheles gambiaecomplex from west Africa and further evidence for reproductive isolation of the Mopti form Parassitologia 1999 41 319 322 10697876

[B9] Dabire KR Diabate A Namountougou M Toe KH Ouari A Kengne P Bass C Baldet T Distribution of pyrethroid and DDT resistance and the L1014F kdr mutation inAnopheles gambiaes.l. from Burkina Faso (West Africa) Trans R Soc Trop Med Hyg 2009 103 1113 1120 10.1016/j.trstmh.2009.01.008 19246066

[B10] Diabate A Baldet T Chandre F Guiguemde RT Brengues C Guillet P Hemingway J Hougard JM First report of the kdr mutation in Anopheles gambiae M form from Burkina Faso, west Africa Parassitologia 2002 44 157 158 12701378

[B11] Collins FH Mendez MA Rasmussen MO Mehaffey PC Besansky NJ Finnerty V A ribosomal RNA gene probe differentiates member species of the Anopheles gambiae complex Am J Trop Med Hyg 1987 37 37 41 2886070 10.4269/ajtmh.1987.37.37

[B12] Scott JA Brogdon WG Collins FH Identification of single specimens of the Anopheles gambiae complex by the polymerase chain reaction Am J Trop Med Hyg 1993 49 520 529 8214283 10.4269/ajtmh.1993.49.520

[B13] Favia G Lanfrancotti A Spanos L Siden-Kiamos I Louis C Molecular characterization of ribosomal DNA polymorphisms discriminating among chromosomal forms of Anopheles gambiae s.s Insect Mol Biol 2001 10 19 23 10.1046/j.1365-2583.2001.00236.x 11240633

[B14] Santolamazza F Mancini E Simard F Qi Y Tu Z della Torre A Insertion polymorphisms of SINE200 retrotransposons within speciation islands ofAnopheles gambiaemolecular forms Malar J 2008 7 163 10.1186/1475-2875-7-163 18724871 PMC2546427

[B15] Bass C Nikou D Donnelly MJ Williamson MS Ranson H Ball A Vontas J Field LM Detection of knockdown resistance (kdr) mutations in Anopheles gambiae: a comparison of two new high-throughput assays with existing methods Malar J 2007 6 111 10.1186/1475-2875-6-111 17697325 PMC1971715

[B16] Martinez-Torres D Chandre F Williamson MS Darriet F Berge JB Devonshire AL Guillet P Pasteur N Pauron D Molecular characterization of pyrethroid knockdown resistance (kdr) in the major malaria vectorAnopheles gambiaes.s Insect Mol Biol 1998 7 179 184 10.1046/j.1365-2583.1998.72062.x 9535162

[B17] Ranson H Jensen B Vulule JM Wang X Hemingway J Collins FH Identification of a point mutation in the voltage-gated sodium channel gene of Kenyan Anopheles gambiae associated with resistance to DDT and pyrethroids Insect Mol Biol 2000 9 491 497 10.1046/j.1365-2583.2000.00209.x 11029667

[B18] Weill M Malcolm C Chandre F Mogensen K Berthomieu A Marquine M Raymond M The unique mutation in ace-1 giving high insecticide resistance is easily detectable in mosquito vectors Insect Mol Biol 2004 13 1 7 10.1111/j.1365-2583.2004.00452.x 14728661

[B19] Wirtz RA Burkot TR Graves PM Andre RG Field evaluation of enzyme-linked immunosorbent assays for Plasmodium falciparum and Plasmodium vivax sporozoites in mosquitoes (Diptera: Culicidae) from Papua New Guinea J Med Entomol 1987 24 433 437 3305949 10.1093/jmedent/24.4.433

[B20] WHO Tests procedures for insecticide resistance monitoring in malaria vector, bioefficacy and persistence of insecticides on treated surfaces WHO/CDS/CPC/MAL/98 1998 12 43

[B21] Riehle MM Guelbeogo WM Gneme A Eiglmeier K Holm I Bischoff E Garnier T Snyder GM Li X Markianos K Sagnon N Vernick KD A cryptic subgroup of Anopheles gambiae is highly susceptible to human malaria parasites Science 2011 331 596 598 10.1126/science.1196759 21292978 PMC3065189

[B22] Djegbe I Boussari O Sidick A Martin T Ranson H Chandre F Akogbeto M Corbel V Dynamics of insecticide resistance in malaria vectors in Benin: first evidence of the presence of L1014S kdr mutation in Anopheles gambiae from West Africa Malar J 2011 10 261 10.1186/1475-2875-10-261 21910856 PMC3179749

[B23] Kawada H Dida GO Ohashi K Komagata O Kasai S Tomita T Sonye G Maekawa Y Mwatele C Njenga SM Mwandawiro C Minakawa N Takagi M Multimodal pyrethroid resistance in malaria vectors,Anopheles gambiaes.s.,Anopheles arabiensis, andAnopheles funestuss.s. in western Kenya PLoS One 2011 6 e22574 10.1371/journal.pone.0022574 21853038 PMC3154902

[B24] Protopopoff N Verhaeghen K Van Bortel W Roelants P Marcotty T Baza D D'Alessandro U Coosemans M A significant increase in kdr in Anopheles gambiae is associated with an intensive vector control intervention in Burundi highlands Trop Med Int Health 2008 13 1479 1487 10.1111/j.1365-3156.2008.02164.x 18983277

[B25] Verhaeghen K Bortel WV Roelants P Okello PE Talisuna A Coosemans M Spatio-temporal patterns in kdr frequency in permethrin and DDT resistantAnopheles gambiaes.s. from Uganda Am J Trop Med Hyg 2010 82 566 573 10.4269/ajtmh.2010.08-0668 20348500 PMC2844549

[B26] Santolamazza F Calzetta M Etang J Barrese E Dia I Caccone A Donnelly MJ Petrarca V Simard F Pinto J della Torre A Distribution of knock-down resistance mutations inAnopheles gambiaemolecular forms in west and west-central Africa Malar J 2008 7 74 10.1186/1475-2875-7-74 18445265 PMC2405802

[B27] Nwane P Etang J Chouasmall yi UM Toto JC Mimpfoundi R Simard F Kdr-based insecticide resistance inAnopheles gambiaes.s populations in BMC Res Notes 2011 4 463 10.1186/1756-0500-4-463 22035176 PMC3221647

[B28] Mathias DK Ochomo E Atieli F Ombok M Nabie Bayoh M Olang G Muhia D Kamau L Vulule JM Hamel MJ Hawley WA Walker ED Gimnig JE Spatial and temporal variation in the kdr allele L1014S inAnopheles gambiaes.s. and phenotypic variability in susceptibility to insecticides in Western Kenya Malar J 2011 10 10 10.1186/1475-2875-10-10 21235783 PMC3029224

[B29] Burton MJ Mellor IR Duce IR Davies TG Field LM Williamson MS Differential resistance of insect sodium channels with kdr mutations to deltamethrin, permethrin and DDT Insect Biochem Mol Biol 2011 41 723 732 10.1016/j.ibmb.2011.05.004 21640822

[B30] Jones CM Sanou A Guelbeogo WM Sagnon N Johnson PC Ranson H Aging partially restores the efficacy of malaria vector control in insecticide-resistant populations of Anopheles gambiae s.l. from Burkina Faso Malar J 2012 11 24 10.1186/1475-2875-11-24 22269002 PMC3312828

