# Supplementary material for: Three years of insecticide resistance monitoring in Anopheles gambiae in Burkina Faso: resistance on the rise?
Source: Malar J. 2012 Jul 16;11:232. doi: 10.1186/1475-2875-11-232 (PMC3489511; doi:10.1186/1475-2875-11-232)
Supplement: Additional file 1 — Pyrethroid bio-assay results for Anopheles gambiae s.l. in four localities after exposure to PBO 4%. An. gambiae mosquitoes, collected from four sentinel sites in 2010 have been exposed to PBO 4% for xxmin, prior to an exposure deltamethrin Permethrin for 1 hour. This table presents the mortality and standard error for each insecticide and locality. [file 1475-2875-11-232-S1.doc]

Additional file 2

| Insecticide | Locality | Mortality | Standard error |
| --- | --- | --- | --- |
| Permethrin | Goundry | 98.11% | 0.02 |
|  | Koupela | 92.27% | 0.05 |
|  | Kuinima | 91.29% | 0.06 |
|  | Soumousso | 94.23% | 0.04 |
| Deltamethrin | Goundry | 100.00% | - |
|  | Koupela | 100.00% | - |
|  | Kuinima | - | - |
|  | Soumousso | - | - |
