# Supplementary material for: Three years of insecticide resistance monitoring in Anopheles gambiae in Burkina Faso: resistance on the rise?
Source: Malar J. 2012 Jul 16;11:232. doi: 10.1186/1475-2875-11-232 (PMC3489511; doi:10.1186/1475-2875-11-232)
Supplement: Additional file 2 — Species composition of An gambiae s.l between the mid and end of the malaria transmission season. The data provided represent the statistical comparison of An. gambiae complex species and molecular forms composition between the mid and end of malaria transmission season during the three years of the study in the four localities. An. gambiae M form (M), An. gambiae S form (S), hybrid of S and M form, (H) and An. arabiensis (B) proportions are given by collection round, the mean and confidence limits (CL) of the proportion are given for each species and locality. The total number of mosquitoes PCR-tested is given for each round (R1, R2) and the P value comparing species composition between rounds is given. [file 1475-2875-11-232-S2.rtf]

Additional file 1:  Species composition of An gambiae sl between the mid and end of the malaria transmission season 
		2008			2009			2010			
Locality	Species	R1	R2		R1	R2		R1	R2	Mean (CL)	
Goundry	B	0.68	0.73		0.73	0.55		0.35	0.38	0.56 (0.43 - 0.71)	
	H	0.09	0		0	0		0	0	0.01 (0.00 - 0.04)	
	M	0.11	0		0.2	0.29		0.25	0.21	0.17 (0.09 - 0.26)	
	S	0.11	0.28		0.08	0.16		0.4	0.41	0.23 (0.12 - 0.35)	
Total		44	40		40	38		204	259		
P-value		0.007			0.303			0.733			
											
Koupela	B	0.6	0.7		0.58	0.82		0.39	0.34	0.56 (0.42 - 0.71)	
	H	0	0		0	0.03		0.03	0	0.01 (0.00 - 0.02)	
	M	0.33	0.15		0.37	0.05		0.46	0.23	0.26 (0.14 - 0.39)	
	S	0.07	0.15		0.05	0.1		0.13	0.43	0.15 (0.05 - 0.26)	
Total		50	32		38	39		232	264		
P-value		0.122			0.002			0.000			
											
Kuinima	B	0.76	0.2		0.49	0.41		0.27	0.27	0.39 (0.24 - 0.55)	
	H	0	0		0.03	0		0	0.01	0.01 (0.00 - 0.01)	
	M	0	0.08		0	0.31		0.11	0.11	0.1 (0.01 - 0.19)	
	S	0.24	0.73		0.49	0.28		0.63	0.61	0.48 (0.33 - 0.65)	
Total		40	40		37	39		267	271		
P-value		0.000			0.000			0.950			
											
Soumousso	B	0.05	0.5		0.33	0.49		0.32	0.25	0.31 (0.18 - 0.46)	
	H	0.03	0		0	0		0	0	0.00 (0.00 - 0.01)	
	M	0	0.1		0.11	0.15		0.22	0.2	0.13 (0.07 - 0.20)	
	S	0.93	0.4		0.56	0.36		0.46	0.55	0.53 (0.39 - 0.69)	
Total		40	40		36	39		245	270		
P-value		0.000			0.239			0.233			
